# The Effects of Job Quality on the Health of Wage Workers: Congruence between the Hard and Soft Job Quality

**DOI:** 10.1016/j.shaw.2022.10.003

**Published:** 2022-10-18

**Authors:** KonShik Kim

**Affiliations:** Department of Business Administration, Kyung Hee University, Seoul, Republic of Korea

**Keywords:** Formative model, Job quality, KWCS, Response surface analysis, Workers' health

## Abstract

**Background:**

This study analyzes the linear and non-linear effects of the hard and soft dimensions of job quality on the overall health of wage workers. It also examines the congruence or fit between the hard and soft job quality on the overall health of wage workers.

**Methods:**

This study measured thirty indicators that constitute job quality and reduced the indicators into twelve sub-dimensions of job quality using reflective factor analysis. In addition, this study derived two dimensions of job quality from the twelve subdimensions, namely the hard and soft job quality using formative factor analysis. This paper applied the response surface analysis to analyze the congruence effect between the two dimensions of job quality.

**Results:**

A logarithmic relationship was found between the dimension of hard job quality and the worker’s overall health. This study also verified that the congruence effect between the two dimensions of job quality does not exist, and the combined effect of job quality is lower when the two dimensions of job quality are at the same level than the effect when either level of job quality is high or low.

**Conclusions:**

Although hard and soft job quality has independent positive effects on the overall health of wage workers, the two dimensions of job quality are not congruent or not in harmony with each other. This incongruence between hard and soft job quality, together with a higher impact of hard job quality, suggests that the role of soft job quality on overall health is relatively limited.

## 1 Introduction

Job quality has been studied as an important factor for job stress, physical, and mental health, thereby increasing life satisfaction or quality of life [[1], [2], [3], [4]]. Policymakers and international organizations have increased awareness of job quality over the past two decades, and research to define and measure job quality has resulted in the development of several international frameworks on the quality of jobs [[5], [6], [7]]. These frameworks have expanded their role as a basis for establishing better labor relations and policies, proposing a wide range of information for industrial and international comparisons. The European Foundation for Improving Living and Working Conditions (EWCS), e.g., developed a framework for measuring job quality in 33 European countries based on the results of the 5th EWCS. This framework consists of seven dimensions: physical environment, labor intensity, quality of working hours, social environment, skills and discretion or autonomy, job prospects, and income. The effect of certain attributes of job quality on the health of wage workers, such as the physical environment of jobs and working hours, has been studied considerably [[8], [9], [10]]. However, few studies have analyzed the impact of job quality on health with a comprehensive index to examine the role of job quality as a whole.

Various attributes or indicators of job quality can be organized into several conceptual and abstract dimensions, and distinguishing job quality into two contrasting dimensions such as hard and soft can be a simple but effective method to analyze the impact of job quality [11]. This dimensional classification or mapping can be helpful when linking the job quality of employees with general management issues to compare and select more effective measures for not only the effectiveness and performance at the organizational level but also the health and well-being at the individual level [12,13]. However, few studies have tried to reduce various indicators of job quality into hard and soft dimensions and analyze the effect of each dimension on the health of wage workers. Adapting the job quality frameworks of previous studies [5,7,[11], [12], [13]], this study defined that the hard dimension of job quality encompasses the elements in terms of physical and contractual working conditions including the physical working environment, working hours, and intensity of work. The soft dimension consists of elements related to social and cultural aspects in the working environment including social support and discretion in the workplace, job prospects, and earnings. Unlike previous research, however, this study examines the dimensionality of job quality using factor analysis, suggesting that numerous job quality indicators can be reduced to higher dimensions of the hard and soft job quality.

Meanwhile, both the hard and soft dimensions of job quality represent the contrasting aspects and conditions of a good job that is not mutually substitutable and congruent to each other. Congruence, in social science research, refers to the degree to which business elements such as job, people, structure, and culture are harmonized or consistent in an organization [14]. The entire organization as a system works efficiently and effectively when these elements of management are consistently matched and fit with each other to support and promote the performance of an organization. Applying this concept of consistency or optimal combination to the concept of job quality, it can be assumed to have a greater positive effect on the health of wage workers if the two dimensions of job quality are implemented together with higher congruence. However, studies on the existence and the effect of congruence between hard and soft dimensions of job quality are extremely rare, failing to provide opportunities to understand the interaction between two dimensions of job quality.

The purpose of this study is threefold. First, it is to investigate the indicators of the job quality of wage workers using raw data from the Institute for Occupational Safety and Health in Korea and to show whether these indicators can be statistically summarized into the hard and hard dimensions. Second, this study will analyze the linear and non-linear effects of the hard and soft dimensions of job quality on the health of wage workers. Third, it will examine the congruence or fit between hard and soft job quality on the overall health of wage workers. By analyzing the relationship between the hard and soft dimensions of job quality from the perspective of congruence, the interactive effect of hard and soft dimensions of job quality on the overall health of wage workers can be more accurately explained.

## 2 Materials and methods

This study uses the survey data from the 5th Korean Working Conditions Survey (KWCS) funded by the Occupational Safety and Health Research Institute (OSHRI) of South Korea. This survey sampled 50,205 workers in 2017 from a population consisting of wage workers, non-wage workers, and self-employed workers. Excluding self-employed and non-wage workers, this study used only the samples of wage workers and the final number of samples was 29,818 after excluding observations with missing variables defined in this study.

Following the framework that proposes indicators for measuring seven dimensions, this study established the hierarchical layers or structures based on the questionnaire items of the KWCS that were designed to share questionnaires with EWCS. In the Appendix I, the first layer consists of seven dimensions, the second layer consists of twelve sub-dimensions, the third layer consists of thirty indicators, and the fourth layer consists of eighty-seven survey questionnaire items. The indicators that have more than one questionnaire item were represented with the ordinal reliability coefficients which all have 0.7 or greater value enough to justify reliability [15]. Appendix II showed the result of the factor structure of indicators and factor loadings with the reliability and validity statistics for sub-dimensions of job quality. The factor loadings of all indicators exceed 0.6, all the composite reliability coefficients for each sub-dimension exceed 0.7, and all the average variances extracted (AVEs) for each sub-dimension also exceed 0.5. These results confirmed that the indicators compose the constructs of each sub-dimension with good reliability and convergent validity. Appendix III exhibited the correlations among sub-dimensions versus the square roots of AVEs of sub-dimensions to examine the discriminant validity among sub-dimensions. The table indicated that the square roots of AVEs shown on the diagonal are greater than the off-diagonal elements in the corresponding rows and columns, demonstrating adequate discriminant validity for all sub-dimensions of job quality. It is noted that the sub-dimension of earnings is not included in Appendix II and Appendix III because it has only one indicator.

All indicators of sub-dimensions were normalized and averaged to build the sub-dimension scores and Table 1 summarizes the sub-dimension scores of job quality with means and standard deviations.

**Table 1:** Sub-dimensions and indicators of job quality dimensions, health, and socioeconomic status with summary statistics∗^,^†

Dimension	Sub-dimension	Indicator	Mean	Standard deviation
Hard job quality	Physical environment	Posture-related risks, ambient risks, biological and chemical risks.	0.793	0.135
	Work demands and pace	Quantitative demands, pace determinants and interdependency, emotional demands	0.713	0.181
	Emotional demands	Interpersonal stress, Client aggression, Emotional disturbance	0.717	0.185
	Work duration	Duration, atypical working time	0.817	0.203
	Working time arrangements	Setting working time arrangements, informing working time changes, flexibility in arrangements	0.882	0.155
	Protection	Adverse social behavior, discrimination	0.648	0.149

Soft job quality	Earnings	Normalized average monthly wage or income	0.161	0.104
	Social support	Social support, intrinsic aspects of work	0.980	0.059
	Work discretion	Cognitive dimension, decision latitude, organizational participation	0.439	0.217
	Training	Training effectiveness, days of training provided by employer, days of on-the-job training	0.156	0.240
	Career prospects	Compensation, job prospects, motivation	0.579	0.163
	Job security	Average tenure, employment contract period, employment status	0.828	0.271

Overall health		The level of risk to your health or safety because of work	1.896	0.306
		The degree of negative effect of work on your health	2.515	0.715
		Subjective assessment of overall health	3.796	0.659
		Longstanding health problems	1.957	0.203
		Number of physical health problems	0.908	0.144

Socioeconomic status		Household income to make ends meet	3.773	1.076
		Number of years of education completed	13.144	3.082
		Occupational prestige based on International SEI	39.204	14.153
		Relative income based on age, sex, and occupation	−0.009	0.088

∗ All indicators were normalized to [0,1] and averaged to construct sub-dimensions of job quality.

† The factor structure between sub-dimensions and indicators was examined and the reliability and validity of sub-dimensions of job quality was confirmed. See Appendix I, II, and III, Appendix II, Appendix III.

Instead of using a reflective model to analyze factors affecting the overall health of wage workers and job quality, this study used a formative model to examine the multi-dimensional structure of job quality [16]. This is because the sub-dimensions of job quality or overall health represent many dimensional areas, each sub-dimension collectively constitutes job quality, sub-dimensions such as physical environment cannot be substituted for other sub-dimensions of job quality, and each sub-dimension is not assumed to be correlated with other sub-dimensions [17]. Meanwhile, this study used the partial least squares (PLS) method to analyze the factor structure with a formative model, assessing the reliability and validity of sub-dimensions within the framework of the PLS method.

Table 2 shows the minimum and maximum values, regression coefficients or indicator weights, variance inflation factors (VIF), and effect sizes measured by f-square coefficients for each sub-dimension of job quality. This study first investigated the factor structure with the assumption that the seven sub-dimensions of the physical environment, work demands and pace, emotional demands, work duration, working time arrangements, protection, and earnings consist of the hard dimension of job quality. In addition, the five sub-dimensions of social support, work discretion, training, career prospects, and job security were assumed to construct the soft dimension of job quality and test whether job quality can be constructed as two dimensions with twelve sub-dimensions that this study established based on the factor analysis results as explained above. The result of this initial structural assumption showed that the indicator weight of the sub-dimension of earnings assigned to the hard dimension of job quality was −0.052 with a negative value and effect size of 0.004, meaning that the earning sub-dimension cannot be the component of the hard job quality dimension. Thus, this sub-dimension of earnings was reassigned to the soft dimension of job quality and the result showed that the indicator weight changed to 0.271 and the effect size also increased to 0.161. These findings verified that the sub-dimension of earnings should be the component of soft job quality and the content and convergent validity of the dimensional structure and twelve components. Table 2 shows the final results that all sub-dimensional indicators have significant relationships with hard or soft dimensions of job quality at the *p* < 0.001 level, showing the reliability and validity of each sub-dimension of job quality [18]. The values of all VIF are less than 2.0, satisfying strict criteria of multicollinearity and confirming that there was no problem due to overlaps between items [19]. The effect sizes of all the sub-dimensions were greater than 0.24, confirming that all the sub-dimensions have enough practical significance [20]. The discriminant validity of hard and soft job quality was evaluated using the correlation coefficients between these variables and overall health. Table 3 showed that the correlation coefficients between those were 0.294, 0.178, and −0.078, respectively. The upper limit of the 95% confidence interval of the largest correlation coefficients was 0.304, much smaller than the general threshold of 0.8 [21]. This result verified that these variables are not highly correlated to each other and demonstrated the discriminant validity between overall health and hard and soft job quality dimensions. All sub-dimensions of hard and soft job quality were normalized and averaged to form variables representing two dimensions of job quality with values ranging from 0 to 1.

**Table 2:** Factor analysis results on hard and soft job quality, health, and SEI using formative model∗

Dimension	Sub-dimension or indicator	Weight 1	Weight 2	Weight 3	Weight 4	VIF	Effect size
Hard job quality	Physical environment	0.336∗∗∗				1.173	0.209
	Work demands and pace	0.364∗∗∗				1.220	0.246
	Emotional demands	0.243∗∗∗				1.057	0.109
	Work duration	0.284∗∗∗				1.095	0.149
	Working time arrangements	0.349∗∗∗				1.162	0.225
	Protection	0.184∗∗∗				1.032	0.062

Soft job quality	Earnings		0.271∗∗∗			1.227	0.161
	Social support		0.297∗∗∗			1.331	0.195
	Work discretion		0.286∗∗∗			1.214	0.180
	Training		0.231∗∗∗			1.108	0.117
	Career prospects		0.306∗∗∗			1.313	0.206
	Job security		0.253∗∗∗			1.194	0.140

Overall health	The level of risk to your health or safety because of work			0.315∗∗∗		1.120	0.176
	The degree of negative effect of work on your health			0.206∗∗∗		1.055	0.075
	Subjective assessment of overall health			0.355∗∗∗		1.166	0.224
	Longstanding health problems			0.351∗∗∗		1.141	0.218
	Number of physical health problems			0.416∗∗∗		1.233	0.307

Socioeconomic status index	Household income to make ends meet				0.273∗∗∗	1.055	0.128
	Number of years of education completed				0.484∗∗∗	1.494	0.402
	Occupational prestige				0.477∗∗∗	1.478	0.391
	Relative level of income				0.214∗∗∗	1.032	0.079

∗ All indicator weights of sub-dimensions are significant (*p* < 0.001).

**Table 3:** Descriptive statistics with correlation matrices of the variables∗

Variable		Mean	Std. dev.	A	B	C	D	E	F	G
A	Overall health	0.882	0.090							
B	Hard job quality	0.817	0.087	**0.294**						
C	Soft job quality	0.469	0.117	**0.178**	**−0.078**					
D	Age	4.021	1.282	**−0.254**	**0.053**	**−0.200**				
E	Firm size	3.131	1.372	**0.053**	0.002	**0.321**	**−0.095**			
F	Gender	1.519	0.500	**−0.063**	**0.115**	**−0.187**	**0.042**	**−0.158**		
G	Marital status	0.437	0.496	**−0.024**	**0.017**	**0.051**	**0.090**	−0.010	**0.280**	
H	Socioeconomic status	0.452	0.108	**0.269**	**0.031**	**0.541**	**−0.349**	**0.242**	**−0.109**	**0.102**

∗ Correlation coefficients in bold are *p* < 0.05.

Meanwhile, overall health as the dependent variable was measured using five sub-dimensions or indicators including the level of risk to your health or safety because of work, the degree of negative effects of work on your health, subjective assessment of overall health, the number of longstanding health problems, and the number of physical health problems. The first four sub-dimensions were measured using responses to each corresponding questionnaire item of KWCS. The sub-dimension of the number of physical health problems was built using ten binary questionnaire items asking about physical health problems including backache, muscular pains, headaches, and so on. The ordinal reliability coefficient of this indicator was 0.899, showing strong reliability of measurements. The ten items were normalized and averaged to build the sub-dimension or indicator of the physical health problems. This study applied the formative factor analysis method to construct the overall health index using the five sub-dimensions or indicators explained above because the causality goes from indicators to the factor. Table 2 shows that all sub-dimensions have significant relationships with the overall health at the *p* < 0.001 level, all variance inflation factors are less than 2, and all the effect sizes were greater than 0.2. These statistics confirm the structure that the five sub-dimensions constitute the overall health and showed the reliability and validity of the overall health. All the sub-dimensions of overall health were normalized and averaged to have a range of [0, 1] representing the overall health index.

This study used five control variables that could influence the relationship between job quality and the overall health of wage workers. First, the socioeconomic status of workers was used to control the effects of the social and economic background of households on the relationship between job quality and overall health. This study used four sub-dimensions or indicators to measure socioeconomic status including household income to make ends meet, the number of years of education completed, occupational prestige, and relative level of income. A questionnaire item on a 6-point scale was used that asked the degree of balance between expenditure and income. The number of years of education completed was measured using the survey items asking the education levels ranging from elementary to graduate schools. The International Socioeconomic Index was used as a single index representing occupational prestige for each job category based on the standard occupational classification [22]. The relative income gap was calculated as the difference between individual income and the average income of reference groups. The number of reference groups was 118, which is constructed using the major categories of the standard occupational classification divided by gender and age. This study applied the formative factor analysis method to construct the socioeconomic status index using the indicators explained above because the causality goes from indicators to the factor or socioeconomic status. Table 2 shows that all indicators have significant relationships with the factor at the *p* < 0.001 level, all variance inflation factors are less than 2, and all the effect sizes were greater than 0.07. These statistics confirmed the structure that the four indicators constitute socioeconomic status and verified the reliability and validity of the socioeconomic status index. All the indicators were normalized and averaged to have a range of [0, 1] to build the index of socioeconomic status. Second, the age of workers was used to control for the effect of a generational gap because the evaluation of subjective health, as well as working conditions, can differ from generation to generation. Third, the size of the workplace that workers employed was categorized into six groups and controlled for the size effect of the quality of jobs. Fourth, gender was used as a control variable since women and men can have different preferences for job quality and health. Fifth, marital status was used to control for the different motives and objectives for evaluating job quality and health status.

## 3 Results

### 3.1 Descriptive statistics

The mean and standard deviation of each variable used in this study, as well as their correlation coefficients and significance, are displayed in Table 3. The correlation coefficients between almost all variables were significant, showing that the statistical analysis models of this study are expected to produce potentially significant results and the intended role of control variables can also be expected. Table 4 exhibits the averages of hard and soft job quality, earnings, and overall health by the occupational classification and the type of employment with inequality index including p90/p10 ratio and Gini coefficients. It is noted that this study used the sampling weight of the survey based on a stratified two-stage cluster sampling to generate population estimates, and the number of samples and population estimates by the occupational classification and the type of employment were reported in Table 4. The level of hard job quality was the lowest in craft and equipment workers, and the soft job quality was the lowest in elementary and agricultural workers. The quality of income, which is a sub-dimension of soft job quality, is the lowest in elementary workers and service workers, and the overall health is the lowest in agricultural workers and elementary workers. In addition, there were significant differences in job quality and overall health between regular and daily workers classified by the type of employment status. Meanwhile, the degree of inequality for quality of income measured by p90/p10 ratio and Gini index was the highest, followed by soft job quality, hard job quality, and overall health. In summary, job quality and overall health level differ significantly depending on the job classification and employment status, and the overall health increases as the hard and soft job quality increases.

**Table 4:** Sample distributions and population estimates by hard and soft job quality, earnings, and overall health with inequality index

Type	Description	Sample	Population	Hard job quality	Soft job quality	Monthly earnings (USD)	Overall health
Occupational classification	Managers	135	120,542	0.851	0.604	4,836	0.896
	Professionals and related workers	5,538	4,539,467	0.826	0.525	2,744	0.898
	Clerks	6,238	4,759,592	0.851	0.517	2,797	0.907
	Service workers	3,498	1,954,746	0.796	0.427	1,728	0.868
	Sales workers	4,350	2,008,680	0.794	0.458	2,070	0.892
	Skilled agricultural, forestry and fishery workers	154	78,576	0.844	0.393	1,960	0.828
	Craft and related trades workers	2,431	1,735,194	0.788	0.472	2,597	0.862
	Equipment, machine operating and assembling workers	2,781	2,113,094	0.788	0.484	2,638	0.871
	Elementary workers	4,601	2,261,044	0.828	0.364	1,351	0.846
	Armed forces	92	63,311	0.815	0.564	3,209	0.929
	Total	29,818	19,634,246	0.817	0.469	2,307	0.882

Employment status	Regular workers	22,919	15,855,239	0.815	0.507	2,578	0.888
	Temporary workers	4,900	2,725,908	0.827	0.357	1,338	0.874
	Daily workers	1,999	1,053,099	0.811	0.304	1,591	0.835

Inequality index	p90/p10 ratio			1.315	1.969	4.444	1.239
	Gini coefficient			0.060	0.140	0.326	0.053

The regression analysis with the least square method was used for all analysis models, and the significance tests of the regression coefficient used robust standard errors that alleviate bias due to heterogeneity between observations. The sampling weight of the survey based on a stratified two-stage cluster sampling was used to estimate unbiased and accurate population parameters including regression weights. Meanwhile, the variables of the hard and soft job quality were mean-centered for the convenience of interpretation of the analysis results. The intercept of each dependent variable, thus, means the average of the estimates of each dependent variable when the job quality is at the mean level.

### 3.2 Results of multiple regression analysis

Model H1 of Table 5 is the result of regression analysis of the relationship between the overall health and the two dimensions of job quality. The effects of hard and soft job quality were all significant at the *p* < 0.001 level, verifying that the level of the overall health of wage workers was increased as the hard and soft quality of jobs was increased. The standardized beta coefficients of hard job quality and soft job quality were 0.316 and 0.079, respectively, confirming that the effect of hard job quality was about 4 times stronger than the soft job quality on the overall health of workers. Previous studies, not just limited to the response surface analysis (RSA), often have assumed and analyzed a quadratic or curvilinear relationship between working conditions and outcomes [23,24]. In model H2 of Table 4, the squared term of the hard job quality representing a quadratic relation was significant at the level of *p* < 0.01 in the negative (−) direction. This finding means that the overall health level of workers gradually increases as the hard job quality increases, but the increasing rate decreases following a logarithmic relationship. The square term of the soft job quality was also significant at the *p* < 0.001 level in the negative (−) direction, showing a logarithmic relationship similar to that of hard job quality.

**Table 5:** Regression results for the effects of hard and soft job quality on the overall health

Model	Overall health Model H1		Overall health Model H2		Overall health Model H3	
	Coeff.	Std. Err	Coeff.	Std. Err	Coeff.	Std. Err
Constant	0.918	0.005∗∗∗	0.921	0.005∗∗∗	0.921	0.005∗∗∗
Age	−0.014	0.000∗∗∗	−0.014	0.000∗∗∗	−0.014	0.000∗∗∗
Firm size	−0.003	0.000∗∗∗	−0.002	0.000∗∗∗	−0.002	0.000∗∗∗
Gender	−0.009	0.001∗∗∗	−0.009	0.001∗∗∗	−0.009	0.001∗∗∗
Marital status	−0.006	0.001∗∗∗	−0.007	0.001∗∗∗	−0.007	0.001∗∗∗
Socioeconomic status	0.101	0.007∗∗∗	0.102	0.007∗∗∗	0.102	0.007∗∗∗
Hard job quality	0.336	0.007∗∗∗	0.309	0.007∗∗∗	0.310	0.007∗∗∗
Soft job quality	0.059	0.006∗∗∗	0.060	0.006∗∗∗	0.060	0.006∗∗∗
Hard job qualityˆ2			−0.495	0.061∗∗∗	−0.500	0.061∗∗∗
Soft job qualityˆ2			−0.029	0.035	−0.034	0.035
Hard job quality X Soft job quality					−0.067	0.069

R square	0.2092∗∗∗		0.2130∗∗∗		0.2131∗∗∗	
R square differences			0.0038∗∗∗		0.0001	
Model comparisons			H1 vs H2		H2 vs H3	
*N*	29,818		29,818		29,818	
Estimated size of population	19,634,246		19,634,246		19,634,246	

+*p* < 0.1, ∗*p* < 0.05, ∗∗*p* < 0.01, ∗∗∗*p* < 0.001.

### 3.3 Results of RSA

This paper used the RSA to analyze the main effect of hard and soft job quality and the congruence effect between the two dimensions of job quality [25]. Based on polynomial regression analysis, RSA visualizes the entire spectrum between variables in three-dimensional space using all possible combinations between job quality dimensions [26]. In addition, RSA provides a statistical means of verifying the existence and direction of agreement or fit between the two variables. This study uses the notations, estimation equations, and acceptance criteria for congruence of RSA suggested by Humberg et al. [25] and Edwards [26].

**Fig. 1:**
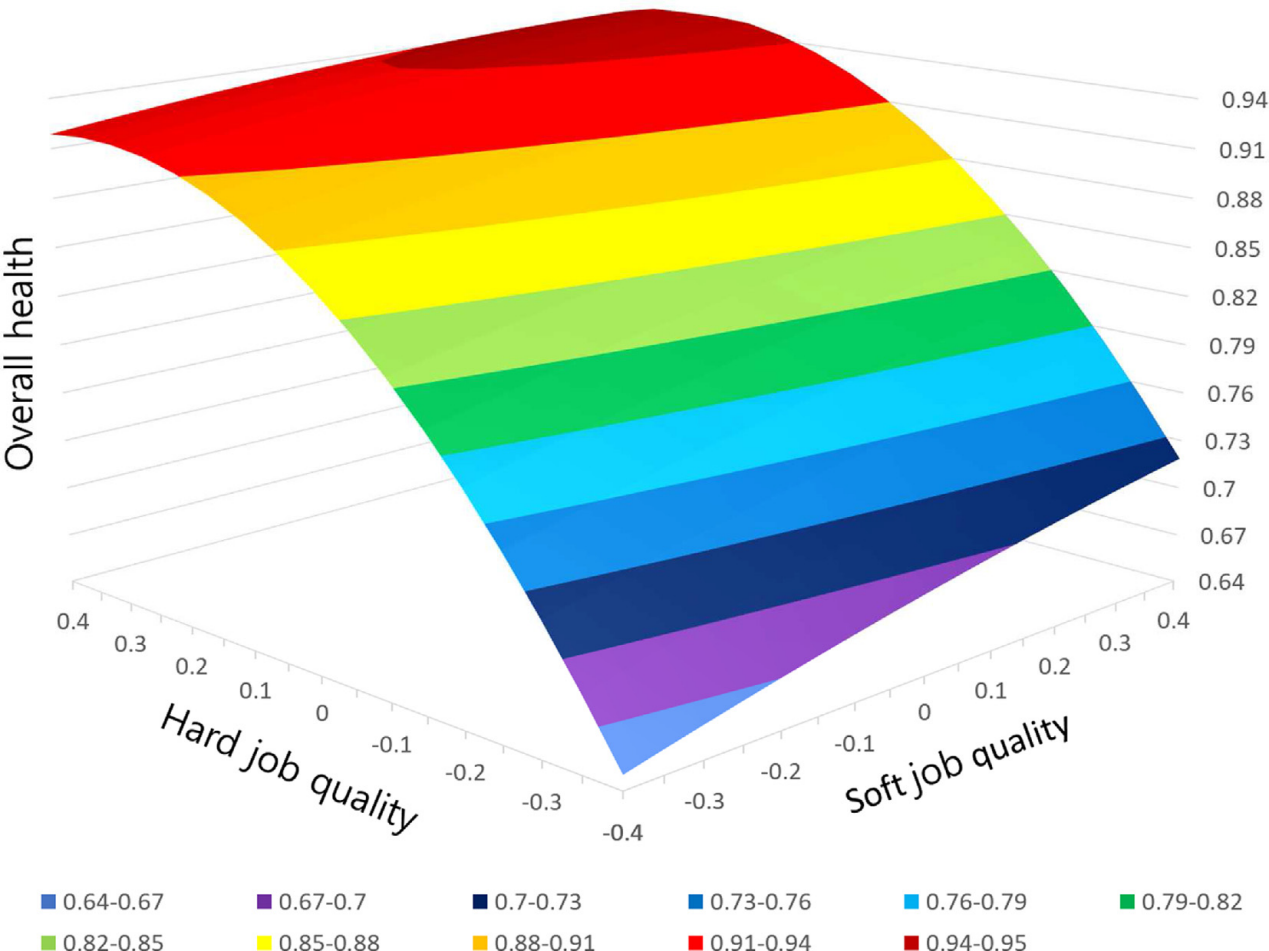
Response surface plot for the effects of the hard and soft job quality on the overall health.

**Fig. 2:**
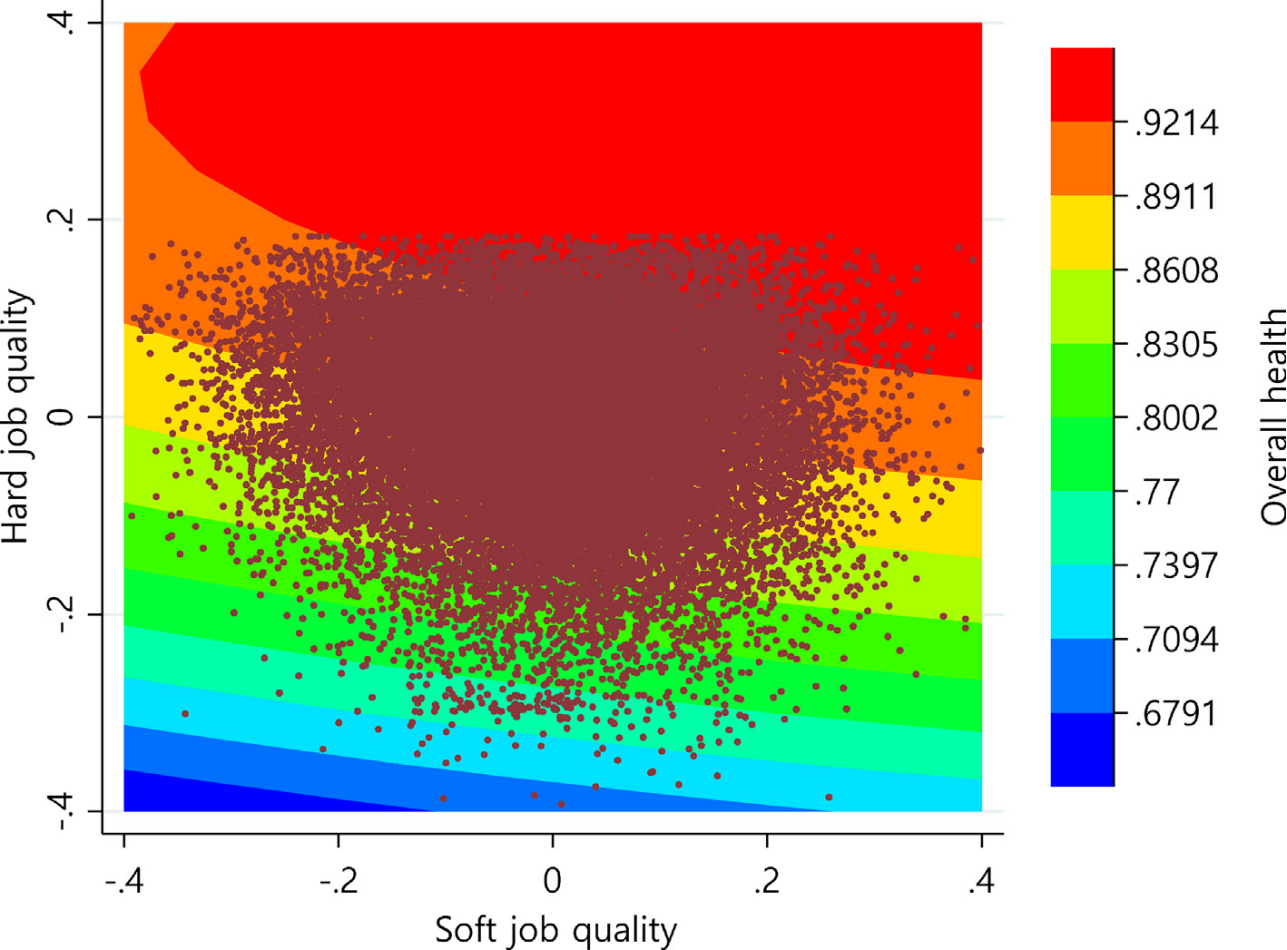
Contour plot for the effects of the hard and soft job quality on the overall health.

The response surface plot in Fig. 1 and the contour plot in Fig. 2 show the interactive effect of hard and soft job quality on the overall health of wage workers. The three-dimensional response surface plot in Fig. 1 shows that the level of hard and soft job quality and the positive impact of job quality on health increases as the response surface goes to the left and right corners of the plot. It means that the more the level of hard and soft job quality increases, the higher the positive impact of job quality on health. The two-dimensional contour map in Fig. 2 shows patterns of relationship between job quality and overall health with the contour lines that connect points where the combination of hard and soft job quality has the same value for overall health. A scatter plot representing the sample value of hard and soft job quality was overlapped on the contour map to indicate the real ranges of job quality dimensions for convenience of interpretation.

Fig. 1, Fig. 2 show that the level of health is not maximized when the levels of the two job quality dimensions are at the highest level. Congruence or fit means that when the values of each job quality are [0, 0], for example, it should have a higher level of health than that when the values of each job quality are [+0.2, −0.2] or [−0.2, +0.2]. However, these figures depict that the level of health is higher when the value of each job quality dimension is [+0.2, −0.2]. In addition, it shows that the congruence effect is even minimized when both levels of job quality are lowest. That is, where the values of the job quality dimensions are [−0.4, −0.4], the level of health is much lower than that where the values of both dimensions are [0, 0].

Table 6 summarizes the results of the analysis with corresponding test statistics, confidence intervals, and standard errors to verify congruence according to the recommendations of Humberg et al. (2019). The consistency effect can be determined only when the test results of p10, p11, a4, and a3 are ‘effective’ by meeting the acceptance criteria set for each test statistic in Table 6. In addition, the test results of a2 and a1 are used to verify the strict condition of congruence that both slope and curvature were not significant. The null hypothesis that there is a congruence effect or fit between two dimensions of job quality was not supported by synthesizing test results and statistics for congruence.

**Table 6:** Results of the statistical test for congruence hypothesis using surface response analysis

Questions to test the congruence hypothesis	Notation and description of test statistic		Acceptance criteria for congruence hypothesis	Overall health	
				Estimates [C.I.]	Test results
Does the first principal axis match the LOC?	p10	Intercept of the first principal axis	No congruence effect if it is significant	4.347 [−4.306 13.001]	Pass
	p11	Slope of the first principal axis	Congruence effect if the confidence interval of p11 includes 1(one)	−13.895 [−41.995 14.205]	Pass
Is the surface above the LOIC an inverted U-shape with vertex at [0,0]?	a4	The curvature of the line of incongruence(discrepancy)	Congruence effect if it is negatively significant	−0.467∗∗	Pass
	a3	The slope of the line of incongruence	No congruence effect if it is significant	0.250∗∗∗	Fail
Is the surface above the LOC constant?	a2	The curvature along the line of perfect agreement	No congruence effect if it is significant	−0.602∗∗∗	Fail
	a1	The slope of the line of perfect agreement	No congruence effect if it is significant	0.370∗∗∗	Fail

+*p* < 0.1, ∗*p* < 0.05, ∗∗*p* < 0.01, ∗∗∗*p* < 0.001.

LOIC: line of incongruence, LOC: Line of congruence.

Meanwhile, Fig. 1, Fig. 2 show that the health level is higher in the back corner of the plot where both hard and soft job quality is higher than in the front corner of the plot. For example, Fig. 2 shows that the level of health is higher in the case where the combination of the two job quality dimensions is [+0.2, +0.2] than in the case of [−0.2, −0.2]. In other words, the ridge of the plot, where the highest agreement is reached between hard and soft job quality, is rising from the front corner to the rear corner. This result means that the level of health increases as the levels of two dimensions of job quality coincide with each other and increase. In the results of the response surface analysis in Table 5, a1 representing the slope of the ridge shows a significant (*p* < 0.001) and positive (+) value. This study, thus, provides statistical evidence that combinations of higher levels of job quality had a greater impact on health than combinations of lower levels.

## 4 Discussion

This study analyzed the effect of hard and soft job quality on the overall health of wage workers and the congruence effect between hard and soft job quality. The results of analyzing survey data of the 2017 KWCS are as follows. First, both hard and soft job quality has a positive and linear impact on the wage worker's overall health. The hard job quality consisting of the physical working environment, labor intensity, and working hours lower the threat to workers' health and safety, thereby reducing health problems. In addition, the soft job quality consisting of skills and autonomy, social working environment, job prospects, and wages also have positive effects on the overall health of wage workers. Meanwhile, the standardized beta coefficient of hard and soft job quality was 0.316 and 0.079, meaning that the effects of hard job quality on health are 4 times greater than those of soft job quality. Second, a logarithmic relationship was found between the dimension of hard job quality and overall health in which the increasing rate of overall health decreases as the level of hard job quality increases. Third, the congruence effect between the two dimensions of job quality does not exist. In other words, the cases where the level of hard and soft job quality matches had smaller effects on health than the cases where one of the job quality dimensions was higher or lower than the other. Fourth, the positive effect of job quality on the health of workers was higher where both levels of job quality were higher than where both levels of job quality were lower.

The theoretical implications of this study on job quality are as follows. First, the quality of jobs can be distinguished as two contrasting dimensions of hard and soft job quality. This study established the hierarchical structures by reducing the eighty-seven survey questionnaire items of the KWCS into thirty indicators and consecutively factored the indicators into the twelve sub-dimensions of job quality. This paper finally identified hard and soft dimensions of job quality using formative factor analysis of the twelve sub-dimensions. In addition, hard job quality consisting of the physical environment, work demands, and working hours has a much greater effect on the overall health of wage workers than the effect of soft job quality including social support, discretion, and training elements. It suggests that focusing on the sub-dimensions of hard job quality can be relatively more efficient measures to improve the overall health of workers.

Second, the logarithmic relationship between the dimension of hard job quality and the overall health of wage workers means that improvement efforts and investments beyond a certain level of hard job quality are less efficient than those below the level of hard job quality. It means that health problems can be solved to some extent if hard job quality increases, but the role of hard job quality has some limitations similar to the role of hygiene factors, making it difficult to expect linear and proportional effects on health.

Third, the effort to increase the level of hard and soft job quality at the same time may be inefficient because the hard and soft job quality are not congruent or fit each other. This study showed that the combined effect of job quality is lower when the two dimensions of job quality are at the same level than the effect when either level of job quality is high or low. This incongruence between hard and soft job quality, together with a higher impact of hard job quality, suggests that the role of soft job quality on overall health is relatively limited. Fourth, the health level of workers is higher when both dimensions of hard and soft job quality are higher than the health level when both dimensions of job quality are lower. These findings imply that rather than just raising the level of hard work quality for health, the level of soft work quality must be raised to some extent to further increase the level of health. In sum, this study found that hard job quality is more effective than soft job quality on the overall health of wage workers in South Korea, and no congruence or fit was found between hard and job quality dimensions. It is noted that the elements of the hard job quality dimension, such as the physical work environment, work demands and pace, and work hours, have been largely controlled by government laws and regulations over decades, but hard or soft job quality has not been publicly measured and no comprehensive policy and action plan in terms of job quality have developed and implemented. More public actions on the working environment and conditions, therefore, are needed not only for the overall health but also for the life satisfaction and well-being of wage workers in this country.

Future research directions are suggested. Further analysis based on longitudinal data on job quality can provide a more causal explanation and in-depth analysis of the degree to which the relationship between job quality and the health of workers. In addition, a future study would benefit from analyzing the structural relationship between job quality and health. Job quality can have an indirect effect on work-life balance and quality of life by mediating health and job engagement. Structural analysis that explains the path or mechanisms from job quality to quality of life can increase the contribution of the study of job quality to the health of workers.

## Conflicts of interest

The author has no conflicts of interest to declare.

## Figures and Tables

**Table:** 

Dimension (EWCS)	Sub-dimension	Indicator	Survey questionnaire item	Mean	Std. Dev.	Alpha∗
Physical environment	Physical environment	Physical hazards	My job involves vibrations from hand tools, machinery etc.	5.933	1.418	0.898
			My job involves noise so loud that you would have to raise your voice to talk to people	5.995	1.226	
			My job involves high temperatures which make you perspire even when not working	5.913	1.318	
			My job involves low temperatures whether indoors or outdoors	5.998	1.208	
		Ergonomics hazards	My job involves tiring or painful positions	5.041	1.657	
			My job involves lifting or moving people	6.235	0.987	
			My job involves carrying or moving heavy loads	5.624	1.334	0.713
			My job involves standing	4.290	1.784	
			My job involves repetitive hand or arm movements	3.922	1.951	
		Biochemical hazards	My job involves breathing in smoke, fumes, powder, or dust, etc.	6.100	1.230	0.928
			My job involves breathing in vapours such as solvents and thinners	6.364	0.893	
			My job involves handling in skin contact with chemical products	6.350	0.909	
			My job involves tobacco smoke from other people	6.238	0.880	
			My job involves handling or being in direct contact with materials which can be infectious	6.434	0.801	
						
Work intensity	Work demands and pace	Quantitative demands	My job involves working at very high speed	4.791	1.705	
			My job involves working to tight deadlines	4.823	1.769	0.724
			How often do you have to interrupt a task you are doing in order to take on an unforeseen task	3.198	0.747	
		Pace determinants and inter-dependency	The pace of work is dependent on the work done by colleagues	0.802	0.398	0.710
			The pace of work is dependent on the direct demands from people such as customers, etc.	0.566	0.496	
			The pace of work is dependent on the numerical production or performance targets	0.855	0.352	
			The pace of work is dependent on the automatic speed of a machine or movement of a product or automatic system	0.926	0.262	
			The pace of work is dependent on the direct control of your boss	0.642	0.479	
	Emotional demands	Interpersonal stress	My job involves dealing directly with people who are not employees at your workplace	4.110	2.254	
		Client aggression	My job involves handling angry clients, customers, patients, pupils etc.	5.786	1.214	
		Emotional disturbance	My job involves being in situations that are emotionally disturbing for you	6.009	0.972	
						
Working time quality	Work duration and atypical working time	Duration	How many hours do you actually work at your workplace in a week or a month	0.713	0.379	0.826
			In the last month, has it happened that you had less than 11 hours between the end of one working day and the start of the next working day?	0.936	0.244	
			How many times a month do you work more than 10 hours a day	0.810	0.393	
		Atypical working time	How many times a month do you work at night	0.907	0.290	0.863
			How many times a month do you work in the evening	0.677	0.467	
			How many times a month do you work on Sundays	0.826	0.379	
			How many times a month do you work on Saturdays	0.565	0.496	
			Do you work for the same number of hours every day	0.838	0.369	
			Do you work for the same number of days every week	0.891	0.311	
			Do you work for the same number of hours every week	0.852	0.355	
			Do you work at fixed starting and finishing times	0.944	0.229	
	Working time arrangements and flexibility	Setting working time arrangements	How are your working time arrangements set?	4.437	1.100	
		Informing working time changes	How long before are you informed about the working time changes?	4.730	0.616	
		Flexibility in arrangements	Arranging to take an hour or two off during working hours to take care of personal or family matters is very easy	4.415	0.860	
						
Social environment	Social support	Social support	Your colleagues or peers help and support you	3.688	0.752	0.715
			Your manager helps and supports you	3.625	0.787	
		Intrinsic aspects of work	Your job gives you the feeling of work well done	3.530	0.753	0.820
			You have the feeling of doing useful work	3.471	0.925	
			You know what's expected of you at work	3.655	0.796	
			You are treated fairly at your workplace	3.540	0.785	
	Protection against adverse social behavior and discrimination	Adverse social behavior	During the course of your work have you been subjected to verbal abuse?	0.952	0.213	0.932
			During the course of your work have you been subjected to unwanted sexual attention?	0.990	0.101	
			During the course of your work have you been subjected to threats?	0.993	0.083	
			During the course of your work have you been subjected to humiliating behavior?	0.966	0.182	
			During the course of your work have you been subjected to physical violence?	0.998	0.049	
			During the course of your work have you been subjected to sexual harassment?	0.994	0.078	
			During the course of your work have you been subjected to bullying/harassment?	0.998	0.040	
		Discrimination	Have you been discriminated by age at work?	1.953	0.212	0.960
			Have you been discriminated by nationality at work?	1.983	0.130	
			Have you been discriminated by race or color at work?	1.988	0.109	
			Have you been discriminated by sex or gender at work?	1.980	0.140	
			Have you been discriminated by religion at work?	1.997	0.059	
			Have you been discriminated by disability at work?	1.980	0.140	
			Have you been discriminated by sexual orientation at work?	1.988	0.110	
			Have you been discriminated by educational background at work?	1.952	0.214	
			Have you been discriminated by hometown at work?	1.987	0.112	
			Have you been discriminated by types of employment status at work?	1.938	0.240	
						
Skills and discretion	Work discretion	Cognitive dimension	Does your job involve solving unforeseen problems on your own?	0.563	0.496	0.712
			Does your job involve complex tasks?	0.349	0.477	
			Does your job involve learning new things?	0.289	0.453	
			How often do you use computers, laptops, smartphones in your work?	3.406	2.176	
			You are able to apply your own ideas in your work	3.372	0.919	
			Does your job involve meeting precise quality standards?	0.288	0.453	
			Does your job involve assessing yourself the quality of your own work?	0.477	0.499	
		Decision latitude	Are you able to choose or change your order of tasks?	0.408	0.491	0.972
			Are you able to choose or change your methods of work?	0.384	0.486	
			Are you able to choose or change your speed or rate of work?	0.379	0.485	
		Organizational participation	You are consulted before objectives are set for your work	3.063	1.000	0.814
			You are involved in improving the work organization or work processes of your department or organization	2.959	1.050	
			You have a say in the choice of your work colleagues	3.018	1.023	
	Training	Training effectiveness	The trainings have helped me improve the way I work.	1.172	1.844	0.989
			The trainings I received increased my job security.	1.110	1.775	
			The trainings I received raised the prospect of future employment.	1.084	1.751	
		Days of training provided by employer	How many days in total did you spend in training paid for or provided by your employer?	2.734	1.437	
		Days of training paid by employee	How many days in total did you spend in training paid by yourself?	0.181	0.385	
						
Prospects	Career prospects	Compensation	Considering all my efforts and achievements in my job, I feel I get paid appropriately	3.332	0.770	
		Job prospects	My job offers good prospects for career advancement	3.182	0.877	
		Motivation	The organization I work for motivates me to give my best job performance	3.456	0.744	
	Job security	Average tenure	Average tenure for workers in month by employment type and status†	75.6	25.9	
		Employment contract period	Did you set an employment contract period when you were hired?	4.291	1.346	
		Employment status	Which of the following is the type of employment under your contract?	2.701	0.586	
						
Earnings	Earnings	Monthly earnings	How much your net monthly earnings from your paid job are? (unit=USD)	2,308	2,203	

∗ The ordinal reliability coefficient suggested by Gadermann et al. [15] was used when the number of questionnaire items for an indicator was more than one and the scales of questionnaire items were binary or ordinal.

† The data of average tenure period were extracted from the webpage of Korean Statistical Information Service (KOSIS) operated by the organization for statistics under the Ministry of Economy and Finance of South Korea. https://kosis.kr/statHtml/statHtml.do?orgId=101&tblId=DT_1DE7079.

**Table:** 

Sub-dimensions Indicators	Physical environment	Work demands and pace	Emotional demands	Work duration	Working time arrangements	Social support	Protection	Work discretion	Training	Career prospects	Job security	Average Variance Extracted (AVE)	Composite Reliability (CR)
Physical hazards	0.907											0.715	0.882
Ergonomics hazards	0.752												
Biochemical hazards	0.870												
Quantitative demands		0.811										0.658	0.794
Pace determinants and inter-dependency		0.811											
Interpersonal stress			0.717									0.534	0.772
Client aggression			0.845										
Emotional disturbance			0.611										
Duration				0.853								0.728	0.843
Atypical working time				0.853									
Setting working time arrangements					0.608							0.513	0.757
Informing working time changes					0.790								
Flexibility in arrangements					0.738								
Social support						0.872						0.760	0.864
Intrinsic aspects of work						0.872							
Adverse social behavior							0.757					0.573	0.729
Discrimination							0.757						
Cognitive dimension								0.818				0.504	0.750
Decision latitude								0.608					
Organizational participation								0.689					
Training effectiveness									0.919			0.699	0.872
Days of training provided by employer									0.905				
Days of on-the-job training									0.658				
Compensation										0.810		0.634	0.838
Job prospects										0.799			
Motivation										0.780			
Average tenure											0.937	0.728	0.887
Employment contract period											0.677		
Employment status											0.921		

**Table:** 

	Indicator	A	B	C	D	E	F	G	H	I	J	K
A	Physical environment	**0.846**										
B	Work demands and pace	0.348	**0.811**									
C	Emotional demands	0.187	0.170	**0.731**								
D	Work duration	0.159	0.131	0.142	**0.853**							
E	Working time arrangements	0.213	0.283	0.211	0.246	**0.716**						
F	Social support	0.039	-0.047	0.001	0.039	0.008	**0.872**					
G	Protection	0.070	0.117	0.133	0.126	0.098	0.066	**0.757**				
H	Work discretion	-0.013	-0.290	-0.075	-0.009	-0.147	0.399	-0.012	**0.710**			
I	Training	0.036	-0.074	-0.051	0.007	-0.068	0.214	-0.075	0.282	**0.836**		
J	Career prospects	0.111	-0.019	-0.009	0.100	0.007	0.436	0.106	0.324	0.195	**0.796**	
K	Job security	0.140	-0.095	-0.062	-0.006	-0.061	0.141	0.013	0.251	0.172	0.230	**0.853**

∗ Square roots of average variances extracted (AVEs) are shown on diagonal.
